# Supplementary material for: Neurobehavioral mechanisms of fear and anxiety in multiple sclerosis
Source: Commun Med (Lond). 2025 Aug 9;5:345. doi: 10.1038/s43856-025-01085-1 (PMC12335550; doi:10.1038/s43856-025-01085-1)
Supplement: Supplementary file 2 — Supplementary Information [file 43856_2025_1085_MOESM2_ESM.pdf]

Neurobehavioral mechanisms of fear and anxiety in multiple sclerosis

SUPPLEMENTARY INFORMATION

Lil Meyer-Arndt[1,2,3,4,5,6], Rebekka Rust[1,2,3,4,7], Judith Bellmann-Strobl[1,2,3,4], Tanja Schmitz-Hübsch[4], Lajos Marko[8], Sofia Forslund[8,9], Michael Scheel[4,10], Stefan M. Gold[11,12,13], Stefan Hetzer[14], Friedemann Paul[1,2,3,4], Martin Weygandt[1,2,3,*]

[1] Max Delbrück Center for Molecular Medicine in the Helmholtz Association, Berlin, Germany
[2] Charité – Universitätsmedizin Berlin, corporate member of Freie Universität Berlin and Humboldt-Universität zu Berlin, Experimental and Clinical Research Center, Berlin, Germany
[3] Experimental and Clinical Research Center, a cooperation between the Max Delbrück Center for Molecular Medicine in the Helmholtz Association and Charité - Universitätsmedizin Berlin, Berlin, Germany
[4] Charité – Universitätsmedizin Berlin, corporate member of Freie Universität Berlin, Humboldt-Universität zu Berlin, and Berlin Institute of Health, NeuroCure Clinical Research Center, Berlin, Germany
[5] Charité – Universitätsmedizin Berlin, corporate member of Freie Universität Berlin, Humboldt-Universität zu Berlin, and Berlin Institute of Health, Department of Neurology and Experimental Neurology, Berlin, Germany
[6] Berlin Institute of Health at Charité –Universitätsmedizin Berlin, BIH Biomedical Innovation Academy, BIH Charité (Junior) (Digital) Clinician Scientist Program, Berlin, Germany
[7] Institute for Immunology, Charité – Universitätsmedizin Berlin Campus Virchow-Klinikum (CVK), Berlin, Germany
[8] Experimental and Clinical Research Center, Charité - Universitätsmedizin Berlin and Max Delbrück Center for Molecular Medicine, Berlin, Germany; German Centre for Cardiovascular Research (DZHK), Berlin, Germany; Berlin Institute of Health, Charité - Universitätsmedizin Berlin, Berlin, Germany; Charité - Universitätsmedizin Berlin, Freie Universität Berlin and Humboldt-Universität zu Berlin, Berlin, Germany
[9] European Molecular Biology Laboratory, Structural and Computational Biology Unit, Heidelberg, Germany
[10] Charité - Universitätsmedizin Berlin, Corporate Member of Freie Universität Berlin, Humboldt-Universität zu Berlin, Department of Neuroradiology, Berlin, Germany
[11] Charité – Universitätsmedizin Berlin, corporate member of Freie Universität Berlin, Humboldt-Universität zu Berlin, and Berlin Institute of Health, Department of Psychiatry and Psychotherapy, Berlin, Germany
[12] Charité – Universitätsmedizin Berlin, corporate member of Freie Universität Berlin, Humboldt-Universität zu Berlin, and Berlin Institute of Health, Department of Psychosomatic Medicine, Berlin, Germany
[13] Institute of Neuroimmunology and Multiple Sclerosis (INIMS), Center for Molecular Neurobiology Hamburg, Universitätsklinikum Hamburg-Eppendorf, Hamburg, Germany
[14] Berlin Center for Advanced Neuroimaging (BCAN), Charité-Universitätsmedizin Berlin, Corporate Member of Freie Universität Berlin and Humboldt-Universität zu Berlin, Berlin, Germany

*Corresponding author:

Martin Weygandt, Robert-Rössle-Straße 1ß, 13125 Berlin, Germany

martin.weygandt@mdc-berlin.de

Supplementary methods

fMRI fear generalization task

In all task stages (pre-acquisition, acquisition, and generalization), the trials were arranged in a pseudo-random sequence with no more than two stimuli of the same type occurring consecutively. Generalization runs were additionally arranged in four blocks to guarantee an even distribution of the four paired presentations of CS+ & US per generalization run. Specifically, each block comprised two trials for each stimulus type except for CS+ & US combinations, which were presented once per block. Further, each of the stimuli was rated four times per pre-acquisition and generalization run and three times in the acquisition run. The sequence of the arrowheads was determined in a pseudorandom fashion for each participant, stage, and run. Starting with the smallest ring having an inner radius of 80 pixels, the radius linearly increased by 40 pixels per ring and thus reached a radius of 240 pixels in the largest one; the length of the inner edges of the square was 160 pixels. The viewing distance (i.e., the distance between the center of the scanner's field of view and the MRI compatible monitor for stimulus presentation) was 1.57 m. Prior to fMRI scanning, each person participated in a training run comprising eight trials and lasting for one minute to familiarize with the basic experimental setup. During this training run, no shocks were applied.

Further, a Digitimer DS7A constant current stimulator using magnetically shielded cable leads grounded through an RF filter was used for shock aplicatioon in the task. Electrodes were attached at the left ankle and a shock calibration procedure was conducted to identify a subject-specific shock intensity level. In this staircase procedure, the intensity level for the fMRI experiment was determined for each participant by applying electric shocks (pulses of 0.5 ms duration with less than 300 volts) of increasing strength starting at a very low level (i.e., 1 mA) up to a level, which the participant perceived as "highly uncomfortable but not yet painful." For PwMSA, the selected average shock intensity was M = 19.5 (SD = 6.5) mA, for PwMSNA it was M = 19.0 (SD = 5.6) mA, and for HPs M = 19.3 (SD = 7.9) mA. The groups did not differ in intensity (PwMSA vs. PwMSNA: t = 0.29, p = 0.771; PwMSA vs. HPs: t = 0.07, p = 0.948; PwMSNA vs. HPs: t = -0.20, p = 0.844). During the fMRI experiment, three pulses of 0.5 ms duration separated by a 50 ms break were applied per US.

MRI sequences

All MR images were acquired with the same 3 Tesla whole-body tomograph (Magnetom Prisma, Siemens, Erlangen, Germany) and a 64-channel head coil. Acquisition of anatomical MRI scans comprised a saggital T1-weighted (T1w; 3-D-Magnetization Prepared Rapid Gradient Echo; MPRAGE) sequence with 208 slices encompassing the entire brain (0.8 mm^3 isotropic voxels; TR = 2400 ms; TE = 2.22 ms; FA = 8°; FOV = 240 × 256 mm^2; matrix size = 300 × 320; 5 min 8 s) and a sagittal FLAIR sequence (208 slices; 0.8 mm^3 isotropic voxels; TR = 6000 ms; TE = 387 ms; TI = 2100 ms; FA = 120°; FOV = 240 × 256 mm^2; matrix size = 300 × 320; 7 min 44 s duration).

Furthermore, functional scans were acquired using a T2*-weighted multi-band Echo-Planar-Imaging (EPI) Blood-Oxygen-Level-Dependent (BOLD) sequence from the Human Connectome Project (26) with 72 axial slices covering the whole brain was acquired (2 × 2 × 2 mm^3 isotropic voxels; TR = 800 ms; TE = 37 ms; flip angle = 52°; FOV = 208 mm × 208 mm; matrix size = 104 × 104; multi-band factor = 8). The sequence for pre-acquisiton / acquisition / generalization runs comprised 495 / 465 / 535 scans and had a duration of 6 min 36 s / 6 min 12 s / 7 min 8 s. In addition, two spin-echo EPI reference volumes with opposing phase encoding directions (anterior to posterior, posterior to anterior) were acquired prior to the first fMRI run with matching readout and geometry for conducting a distortion correction of fMRI scans.

Finally, the DWI data required for this purpose were acquired with a state-of-the-art multi-shell DWI MRI sequence from the Human Connectome Project (26) with 92 axial slices covering the whole brain (1.5 × 1.5 × 1.5 mm^3 isotropic voxels; TR = 3230 ms; TE = 89.2 ms; flip angle = 78°; FOV = 210 · 210 mm^2; matrix size 140 × 140; bandwidth = 1700 Hz/pixel; 2 shells [b = 1500 / 3000 s/mm^2] ; 92 to 93 directions per shell; multi-band factor = 4, phase-encoding direction anterior-to-posterior). Further, we acquired pairs of spin-echo EPI reference volumes with opposing phase-encoding directions (anterior-to-posterior, posterior-to-anterior) prior to the DWI scans with matching readout and geometry for distortion correction of the DWI images.

Processing of anatomical MRI scans

Experienced raters generated lesion masks for each participant under the supervision of a neuroradiologist based on participants' FLAIR images for assisting in a combined spatial normalization and segmentation step described below and to determine the whole-brain lesion volume.

As preparation for the combined spatial normalization and segmentation of T1-weighted MP-RAGE images conducted with SPM12 (Wellcome Trust Centre for Neuroimaging, Institute of Neurology, University College London, London, UK, http://www.fil.ion.ucl.ac.uk/spm), the FLAIR scan of a participant and the derived lesion mask were coregistered to their T1-weighted scan. Afterwards, the T1-weighted scan was non-linearly coregistered to the anatomical standard space defined by the Montreal Neuological Institute (MNI; 1) and segemented into grey matter (GM), white matter (WM) and cerebro-spinal fluid (CSF) using the combined SPM12 algorithm. Voxel coordinates located in lesions according to the coregistered lesion masks were discarded. The segmentation procedure yielded tissue probability maps in the participant-specific/native image space and MNI space. MNI-space images were adjusted for normalization-induced local deformations. Native image space maps were used to calculate the participants' GM fractions (i.e., the number of GM voxels divided by the sum of all intracranial voxels). The deformation fields computed here were used to map the fMRI scans from native to MNI space and to inversely normalize the Neuromorphometric brain atlas to the native image space of the participants' DWI scans. The deformation-corrected probability maps determined in MNI space (together with the coregistered lesion masks) were used for determining the GM group mask.

To determine this mask, each voxel coordinate was assigned to that tissue for which the maximal modulated tissue probability averaged across all 23 HPs was computed in the combined normalization and segmentation step. Coordinates located in lesions according to the coregistered lesion masks and the six direct/closest neighbors of such coordinates were classified as lesion tissue to account for partial voluming. All coordinates classified as GM were entered into the GM group mask.

Statistical analysis

<u>*Behavioral fear generalization*</u>

Quality assurance steps

Before testing our hypothesis that PwMSA overgeneralize fear on a behavioral level by evaluating the risk ratings acquired during the task for all ring-shaped stimuli across the two generalization runs, we applied two quality assurance steps in addition to the risk rating criteria which have already been described in the main text.

Proportion of participants exposed to the smallest or largest ring as the CS+: Second, we verified that the proportion of participants exposed to the smallest or largest ring as the CS+ was similar across groups to affirm that neurobehavioral fear responses were not influenced by the physical properties of stimuli. The results are reported in an individual section below.

Successful fear induction by the task: To characterize fear responses during the pre-acquisition and acquisition stage, we computed the average shock ratings for the 67 participants fulfilling the rating data inclusion criteria applied to the generalization runs described in the main text. From these 67 participants, two participants did not rate the risk of shock during the pre-acquisition stage. For the acquisition stage, rating data of all 67 participants were available. The average shock rating was computed separately for CS+, CS_r- and the CS_s-. Based on these data, we tested whether they differed significantly between conditions per group with an uncorrected significance threshold of $\alpha = 0.05$. The results are reported in an individual section below.

Mathematical characterization of risk ratings

To mathematically characterize the risk ratings for each participant, the independent variable for the logistic regression model was derived from the continuum of ring-shaped stimuli. Specifically, the CS_r-, was coded as 1, followed by the GS3 coded as 2, up to the CS+, which was coded as 5. The dependent variable was determined by linearly mapping the response button codes for the rating to a probability range of 0 to 1 (i.e., minimal → 0, moderate → 0.5, and maximal risk → 1). After the logistic regression model was determined for a given participant, we computed their PI based on the model's regression coefficients as $PI = -b_0 / b_1$.

Group differences in behavioral fear generalization adjusted for fatigue and depression

Due to the frequent association between anxiety and depression as well as fatigue in MS (e.g., 2), we here repeated the test of group differences in behavioral fear generalization conducted in the main text but now included fatigue (i.e., participants MFIS scores) and depression (i.e., BDI-II scores) as additional CNI in the regression model.

Neural substrates of behavioral fear generalization

Adjusting fMRI patterns for CNI

Here, we repeated the whole-brain GM analyses conducted in the main text by using fMRI patterns adjusted for the CNI also considered in the analysis of behavioral fear generalization. In particular, to control for conceivable effects of demographic and disease-related factors, we adjusted the patterns of patients voxel-by-voxel for variance explainable by the average rating time, log-transformed volume of hyperintense lesions, disease duration, disease type, age, sex, use of interferon β and antidepressants, depression (BDI-II) and fatigue (MFIS) using linear regression separately for PwMSA and PwMNSA. The training data of HPs were corrected for the average rating time, sex, age, log-transformed lesion volume, fatigue and depression.

Structural brain connectivity, behavioral fear generalization, and anxiety in MS

Adjusting for CNI

Here we repeated the corresponding analysis presented in the main text but now included the patients' depression (i.e., BDI-II) and fatigue (MFIS) scores as additional CNI into the regression models.

Supplementary results

Statistical analysis

Behavioral fear generalization

Proportion of participants exposed to the smallest or largest ring as the CS+

Testing whether the proportion of participants exposed to the smallest or largest ring as CS+ was similar across groups to affirm that neurobehavioral fear responses were not influenced by the physical properties of stimuli shows that these proportions were highly comparable for all pairs of groups (PwMSA: 7 × largest, 6 × smallest; PwMSNA: 18 × largest, 13 × smallest; HPs: 12 × largest, 11 × smallest; PwMSA vs. PwMSNA: $\chi^2 = 0.07$; $p = 0.797$; PwMSA vs HPs: $\chi^2 = 0.01$; $p = 0.923$; PwMSNA vs. HPs: $\chi^2 = 0.19$; $p = 0.667$).

Successful fear induction by the task

Supplementary Fig. 1 displays the results of this analysis.

Group differences in behavioral fear generalization adjusted for fatigue and depression

Although adjusting for the mentioned covariates slightly attenuated effects, the differences for PwMSA vs PwMSNA was still significant on an $\alpha = 0.1$ level, the difference between PwMSA and HPs remained significant at $\alpha = 0.05$. See Supplementary Fig. 2.

Neural substrates of behavioral fear generalization

Adjusting fMRI patterns for CNI

This analysis yielded highly significant accuracies for both groups, i.e., $r_{adjusted\ [adj.]} = 0.51$ ($p < 5 \cdot 10^{-5}$) for PwMSA and $r_{adj.} = 0.58$ ($p < 5 \cdot 10^{-5}$) for PwMSNA. Neither the difference in accuracy computed based on raw and adjusted data for PwMSA (for $r = 0.59$ vs. $r_{adjusted\ [adj.]} = 0.51$; $Z = 0.84$, $p = 0.404$) nor for PwNSA ($r = 0.58$ vs. $r_{adj.} = 0.58$; $Z = -0.08$, $p = 0.93$) was significant according to two-sided Z-tests (3).

Structural brain connectivity, behavioral fear generalization, and anxiety in MS

Adjusting for CNI

In the analysis linking the connectivity measures to behavioral fear generalization (i.e., the patients PI) additionally correcting for BDI-II and MFIS-scores, the association to the

clustering coefficient of left inferior temporal gyrus remained significant. Given that the Bonferroni-method applied throughout the study is the most conservative among all methods for multiple testing adjustment, and the importance of the region in the fear generalization model of Lissek et al. (4), it might be legitimate to mention that the link between clustering coefficient of left hippocampus and behavoral fear generalization was significant according to the False Discovery Rate method (FDR; 5) according to $\alpha_{FDR} = 0.05$ ($t = 3.56$ $p_{FDR} = 0.048$, $f^2 = 0.42$) when additionally correcting for BDI-II and MFIS. The post-hoc analysis testing for group differences in these clustering coefficients again found that this difference was significant for left inferior temporal gyrus. Supplementary Fig. 3 summarizes the results of this analysis.

Finally, also, when the analysis for group differences was additionally corrected for information processing speed as CNI, the difference remained significant ($t = -1.92$, $p = 0.034$, $f^2 = 0.12$).

Supplementary figures

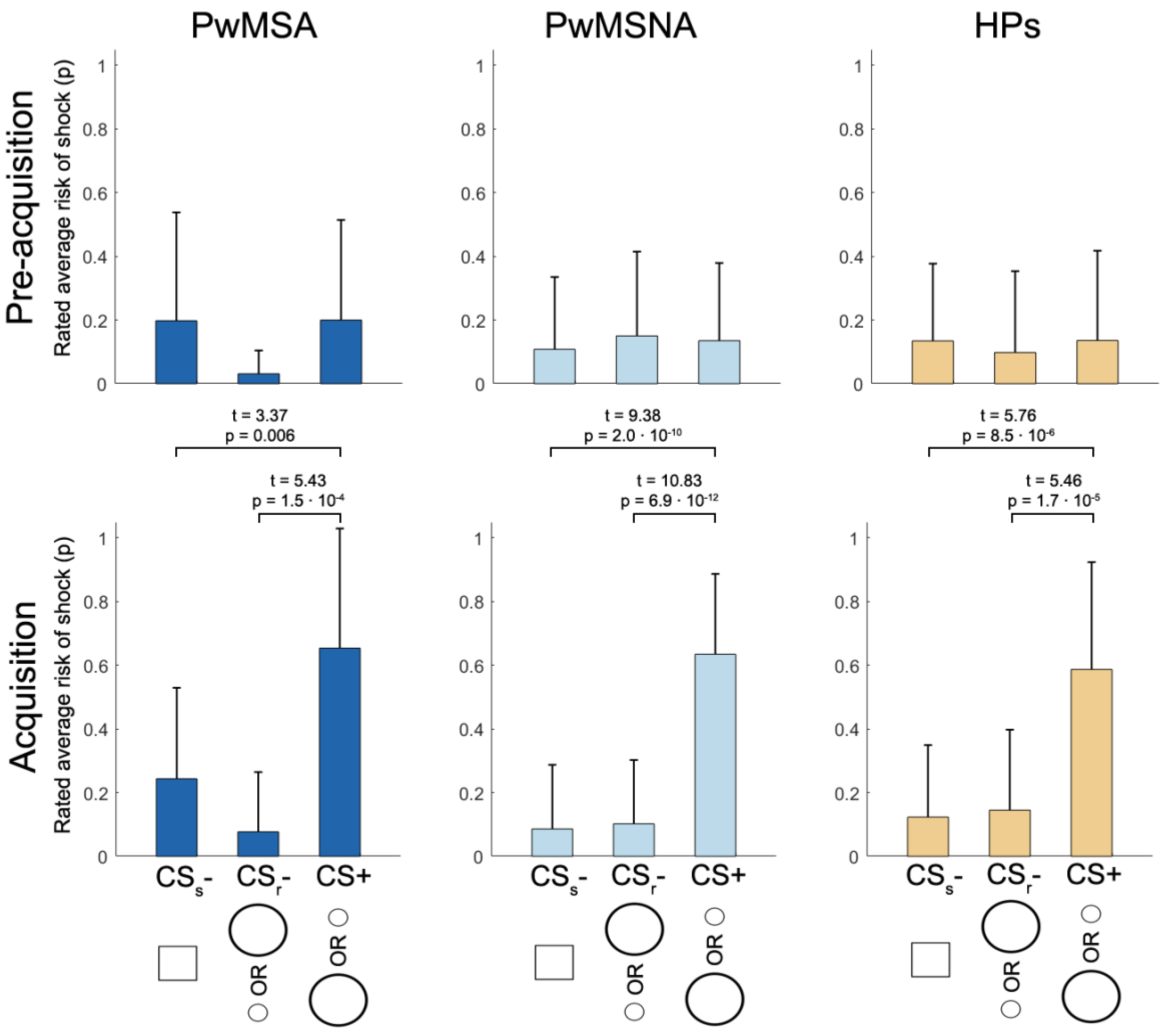

Supplementary Fig. 1. Behavioral fear responses during pre-acquisition and acquisition. The height of the bars depicts the mean and the whiskers the standard deviation of the average rating data separately for each experimental stage, condition and group. Pre-acquisition PwMSA n = 12, PwMSNA n = 30, HPs n = 23. Acquisition PwMSA n = 13, PwMSNA n = 31, HPs n = 23. T-statistics and p-values are reported for significant comparisons.

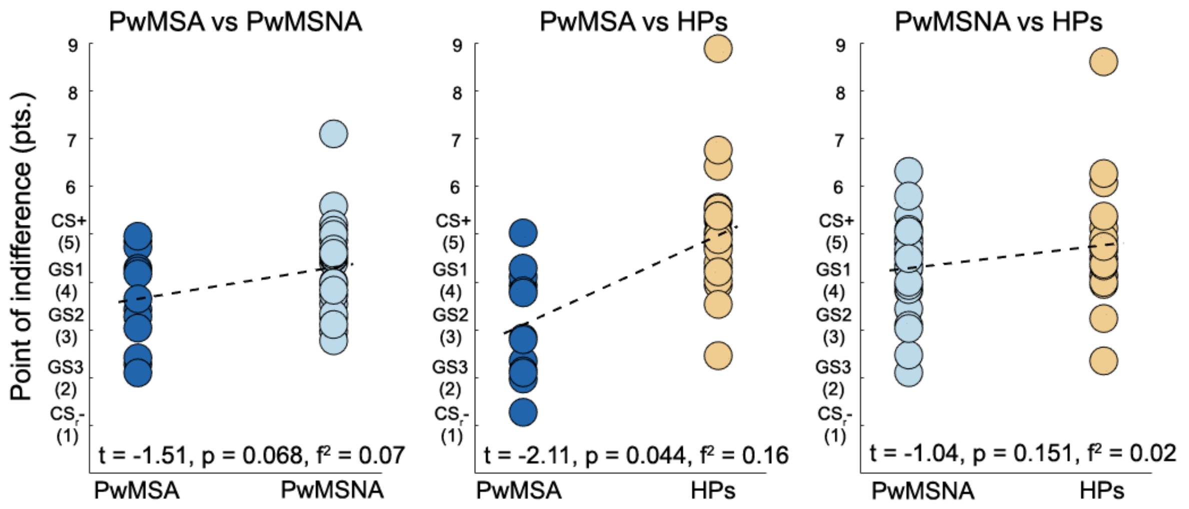

Supplementary Fig. 2. Group differences in behavioral fear generalization controlled for fatigue and depression. For details, see text above and the caption of Fig. 2d.

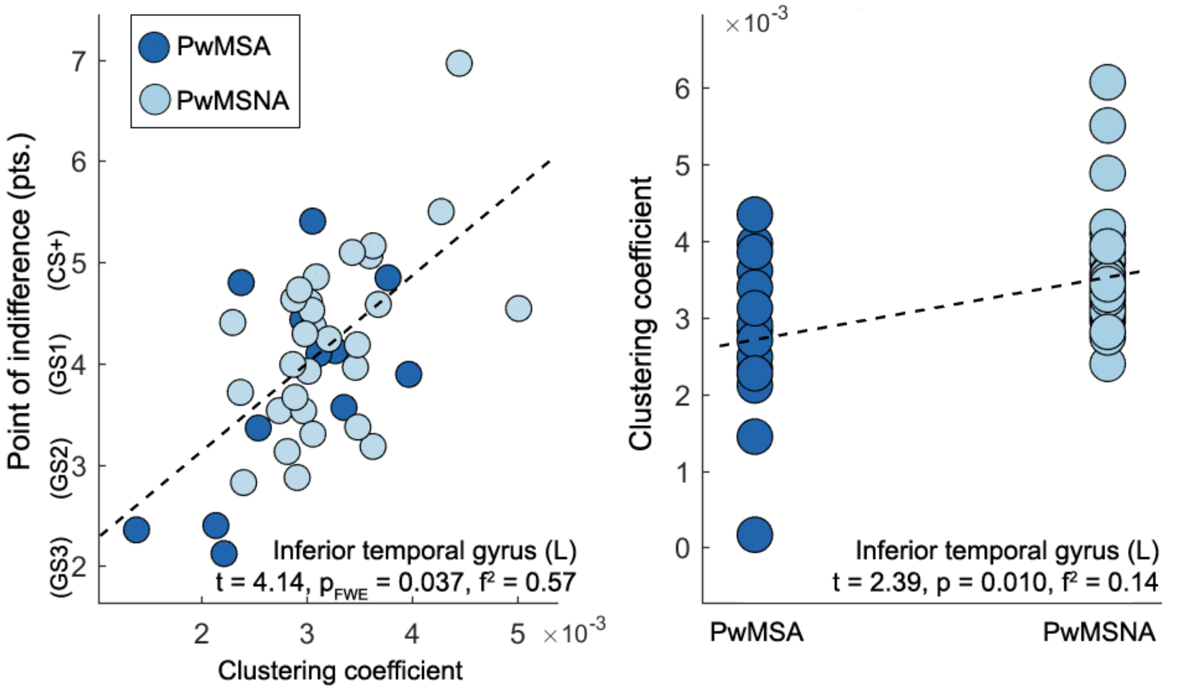

Supplementary Fig. 3. Associations between structural brain connectivity, behavioral fear generalization and anxiety in MS considering the impact of depression and fatigue. For details, see caption of Fig. 5.

Supplementary references

1. Tzourio-Mazoyer N, Landeau B, Papathanassiou D, Crivello F, Etard O, Delcroix N, Mazoyer B, Joliot M. Automated anatomical labeling of activations in SPM using a macroscopic anatomical parcellation of the MNI MRI single-subject brain. Neuroimage. 2002; 15(1):273-89. doi: 10.1006/nimg.2001.0978.
2. AlSaeed S, Aljouee T, Alkhawajah NM, Alarieh R, AlGarni H, Aljarallah S, Ayyash M, Abu-Shaheen A. Fatigue, Depression, and Anxiety Among Ambulating Multiple Sclerosis Patients. Front Immunol. 2022 Mar 29;13:844461. doi: 10.3389/fimmu.2022.844461.
3. Fisher, R. A. (1921). On the "Probable Error" of a Coefficient of Correlation Deduced from a Small Sample. Metron, 1, 3-32.
4. Lissek S. Toward an account of clinical anxiety predicated on basic, neurally mapped mechanisms of Pavlovian fear-learning: the case for conditioned overgeneralization. Depress Anxiety. 2012; 29(4):257-63. doi: 10.1002/da.21922.
5. Benjamini Y & Hochberg Y (1995). Controlling the False Discovery Rate: A Practical and Powerful Approach to Multiple Testing. Journal of the Royal Statistical Society: Series B (Methodological) 57, 289-300.
