# Supplementary material for: Neurobehavioral mechanisms of fear and anxiety in multiple sclerosis
Source: Commun Med (Lond). 2025 Aug 9;5:345. doi: 10.1038/s43856-025-01085-1 (PMC12335550; doi:10.1038/s43856-025-01085-1)
Supplement: Supplementary file 3 — Description of Additional Supplementary Files [file 43856_2025_1085_MOESM3_ESM.pdf]

Supplementary Data 1

Supplementary Table 1. Demographic, clinical and neuroradiographic participant characteristics.

Abbreviations: #, number; BDI – II – Beck Depression Inventory II score; BTK, Bruton Tyrosine Kinase; DD, disease duration; EDSS – Expanded Disability Status Scale; fract., fraction; HSD, high school diploma; IFN-β, β-interferons; Inf. Proc., information processing; MAB, monoclonal antibodies; MD, median; MFIS – Modified Fatigue Impact Scale score; PROG, denotes the presence of a progressive form of the disease; RG, range; RR, relapsing remitting; S1P, sphingosin-1-phosphate analogues; SP, secondary progressive; STAIT-T, State Trait Anxiety Inventory – Trait scale; T2LL, T2-weighted lesion load.

[§]Please note that we report measures of inferential statistics for STAI-T group differences only for the sake of completeness. From a statistical viewpoint, the parameters reported are meaningless as the group assignment was performed based on STAI-T. [*]Information processing reported in milliseconds corresponds to the average time required to rate the perceived risk of shock across trials and participants of a group across both generalization runs. This parameter is reported for the 13 PwMSA, 31 PwMSNA and 23 HPs fulfilling the rating criteria delineated in the Methods section. All other parameters are reported for all 18 PwMSA, 36 PwMSNA, and 23 HPs, the persons for whom either rating and corresponding fMRI data and/or DWI data were available. To test for group differences in EDSS, Wilcoxon rank sum tests were conducted. For all t-tests, two-sided tests were conducted.
