# Supplementary material for: Neurobehavioral mechanisms of fear and anxiety in multiple sclerosis
Source: Commun Med (Lond). 2025 Aug 9;5:345. doi: 10.1038/s43856-025-01085-1 (PMC12335550; doi:10.1038/s43856-025-01085-1)
Supplement: Supplementary file 5 — Reporting Summary [file 43856_2025_1085_MOESM5_ESM.pdf]

Corresponding author(s): DAPR COMMSMED-25-0570-T

Last updated by author(s): 06/13/2025

Reporting Summary

Nature Portfolio wishes to improve the reproducibility of the work that we publish. This form provides structure for consistency and transparency in reporting. For further information on Nature Portfolio policies, see our Editorial Policies and the Editorial Policy Checklist.

Statistics

For all statistical analyses, confirm that the following items are present in the figure legend, table legend, main text, or Methods section.

n/a	Confirmed	
☐	☒	The exact sample size (*n*) for each experimental group/condition, given as a discrete number and unit of measurement
☐	☒	A statement on whether measurements were taken from distinct samples or whether the same sample was measured repeatedly
☐	☒	The statistical test(s) used AND whether they are one- or two-sided<br>*Only common tests should be described solely by name; describe more complex techniques in the Methods section.*
☐	☒	A description of all covariates tested
☐	☒	A description of any assumptions or corrections, such as tests of normality and adjustment for multiple comparisons
☐	☒	A full description of the statistical parameters including central tendency (e.g. means) or other basic estimates (e.g. regression coefficient) AND variation (e.g. standard deviation) or associated estimates of uncertainty (e.g. confidence intervals)
☐	☒	For null hypothesis testing, the test statistic (e.g. F, t, r) with confidence intervals, effect sizes, degrees of freedom and P value noted<br>*Give P values as exact values whenever suitable.*
☒	☐	For Bayesian analysis, information on the choice of priors and Markov chain Monte Carlo settings
☒	☐	For hierarchical and complex designs, identification of the appropriate level for tests and full reporting of outcomes
☐	☒	Estimates of effect sizes (e.g. Cohen's d, Pearson's r), indicating how they were calculated

Our web collection on statistics for biologists contains articles on many of the points above.

Software and code

Policy information about availability of computer code

Data collection	No software was used for data collection.
Data analysis	The analyses of behavioral data were conducted with in-house code utilizing standard functions included in Matlab (2021b). The analyses of fMRI data were conducted with in-house code also written in Matlab or its statistics and machine learning toolbox respectively. Parts of this code make use of SPM12 routines for basic neuroimaging file (i.e., read and write) operations. All in-house routines used for behavioral and fMRI data analyses are available online via the Zenodo data and code repository. Further data processing steps were conducted with freely available neuroimaging software packages. First, preprocessing of structural and functional MRI scans and parts of the preprocessing of the DWI scans were conducted with SPM12 (https://www.fil.ion.ucl.ac.uk/spm/software/spm12/) and FSL (6.0.7.17; https://fsl.fmrib.ox.ac.uk/fsl/docs/#/). Other parts of DWI scan preprocessing were conducted with Mrtrix3 (https://www.mrtrix.org/). The graph-based analyses of the brain's structural connectome were conducted with the Brain Connectivity Toolbox (https://sites.google.com/site/bctnet/; release 3rd of March 2019) for Matlab. These freely available software packages can be received from their respective websites.

For manuscripts utilizing custom algorithms or software that are central to the research but not yet described in published literature, software must be made available to editors and reviewers. We strongly encourage code deposition in a community repository (e.g. GitHub). See the Nature Portfolio guidelines for submitting code & software for further information.

Data

Policy information about availability of data

All manuscripts must include a data availability statement. This statement should provide the following information, where applicable:

- Accession codes, unique identifiers, or web links for publicly available datasets
- A description of any restrictions on data availability
- For clinical datasets or third party data, please ensure that the statement adheres to our policy

The data that supports the findings of this study are subject to confidentiality agreement and the patients have not consented to public release of their data. Thus, only highly processed/aggregated data are available via the Zenodo data and code repository. The following lists the data that is available per analysis conducted:
• Clinical and demographic participant characteristics: The information on the participants' information processing speed depicted in Table 1 is provided.
• Behavioral fear generalization: Data underlying Fig. 2b and c is provided.
• Neural substrates of behavioral fear generalization: The behavioral raw data and run-specific SPM fMRI regression coefficient maps resulting from "fMRI preprocessing and brain activity modeling" is available. These data will allow to fully replicate the statistical parameters depicted in Figure 3 and 4.
• Structural brain connectivity, behavioral fear generalization, and anxiety in MS: Structural connectivity matrices (one of which is depicted in Figure 5b) required to generate the clustering coefficients, betweenness centrality and regional degree (Figure 5c depicts the clustering coefficients for two arbitrarily selected participants) is provided.
• Supplementary analyses:
o Behavioral fear generalization: Proportion of participants exposed to the smallest or largest ring as the CS+: The frequencies underlying this analysis is provided.
o Behavioral fear generalization: Successful fear induction by the task: The raw behavioral data and the code require them to replicate the analyses depicted in Fig. S1 is provided.

Research involving human participants, their data, or biological material

Policy information about studies with human participants or human data. See also policy information about sex, gender (identity/presentation), and sexual orientation and race, ethnicity and racism.

Reporting on sex and gender	Participants of both sexes were included in our study. Sex was assigned based on information from medical records. We did not assess gender identity. 60 participants were female, 17 male.
Reporting on race, ethnicity, or other socially relevant groupings	This study did not collect information on race, ethnicity, or other socially relevant groupings.
Population characteristics	This study reports on participants' age, with a mean of 39.7 years in the PwMSA group, 40.3 years in the PwMSNA group, and 40.8 years in the healthy controls (HPs). In adition to age, we provide detailed clinical data for the MS groups, including current disease-modifying immunotherapy, MS subtype (e.g., relapsing-remitting, secondary progressive), and EDSS scores to assess neurological disability.
Recruitment	Participants were recruited through Charité outpatient clinics and public advertisements, with no apparent sources of recruitment bias.
Ethics oversight	Ethics committee of Charité – Universitätsmedizin Berlin (study number: EA1/209/19)

Note that full information on the approval of the study protocol must also be provided in the manuscript.

Field-specific reporting

Please select the one below that is the best fit for your research. If you are not sure, read the appropriate sections before making your selection.

☒ Life sciences ☐ Behavioural & social sciences ☐ Ecological, evolutionary & environmental sciences

For a reference copy of the document with all sections, see nature.com/documents/nr-reporting-summary-flat.pdf

Life sciences study design

All studies must disclose on these points even when the disclosure is negative.

Sample size	We included 54 individuals with multiple sclerosis (18 with MS and anxiety (PwMSA), and 36 with MS only (PwMSNA)) and 29 healthy controls (HPs) in our study.
Data exclusions	For the behavioral and fMRI analyses, only those participants (i.e., 13 PwMSA, 31 PwMSNA, 23 HPs) who met specific behavioral rating criteria, ensuring full engaged with and understanding of the task, were included. The exclusion procedure is described in detail in the Supplement (see Materials and methods, Statistical analysis, Behavioral fear generalization, Quality assurance steps, Risk rating criteria). DWI analyses related to these behavioral rating data were also constrained to the 13 PwMSA and 31 PwMSNA who met the criteria. However, DWI analyses testing group differences in structural brain connectivity between PwMSA and PwMSNA were conducted using the full sample of 54 MS patients.

Replication	Bootstrapping was performed in the fMRI analysis presented in the main text (evaluating activity across the brain's entire grey matter) to verify that the results were not dependent on the specific distribution of fMRI patterns and labels in our participant sample.
Randomization	The study is observational in nature; therefore, randomization into different treatment groups was neither necessary nor possible.
Blinding	The personnel responsible for acquiring the behavioral and MRI data were blinded to participants' group membership.

Reporting for specific materials, systems and methods

We require information from authors about some types of materials, experimental systems and methods used in many studies. Here, indicate whether each material, system or method listed is relevant to your study. If you are not sure if a list item applies to your research, read the appropriate section before selecting a response.

Materials & experimental systems

n/a	Involved in the study	
☒	☐	Antibodies
☒	☐	Eukaryotic cell lines
☒	☐	Palaeontology and archaeology
☒	☐	Animals and other organisms
☒	☐	Clinical data
☒	☐	Dual use research of concern
☒	☐	Plants

Methods

n/a	Involved in the study	
☒	☐	ChIP-seq
☒	☐	Flow cytometry
☐	☒	MRI-based neuroimaging

Plants

Seed stocks	n/a
Novel plant genotypes	n/a
Authentication	n/a

Magnetic resonance imaging

Experimental design

Design type	fMRI fear generalization task
Design specifications	The fMRI fear generalization task comprised three consecutive stages: pre-acquisition (two runs), acquisition (one run), and generalization (two runs). Across the task, five ring-shaped stimuli (i.e., the CS+, a ring-shaped CS- [“CSr-“] and three GS) and a square-shaped CS- (“CSs-“) were shown (in different compositions; see below). Specifically, during pre-acquisition, all six stimuli were shown in eight trials per run each, and none were paired with an electric shock. During acquisition, the CS+, CSr- and CSs- were shown in 15 trials each and the CS+ co-terminated with an electrical shock (US) in twelve trials. The generalization stage was identical to pre-acquisition, except that it included four additional trials in which the CS+ co-terminated with a shock to prevent fear extinction. Across stages, trials were composed of time bins with two seconds duration. Each trial started with the presentation of a CS or GS (two bins duration) and was followed by an inter-trial-interval (ITI) of one to three bins (average: two bins) duration. Additionally, one of four different arrowheads (a green one pointing up, yellow - left, red - right and blue - down) was shown in the middle of the screen during a bin. The bin-wise structure served two purposes. First, the occurrence of a green arrowhead signaled the participant to rate the perceived risk of shock (ranging from “minimal” over “moderate” to “maximal”) for the currently depicted ring- or square-shaped stimulus as fast as possible with an MRI compatible response box. Second, the participants were instructed to focus the center of the arrowheads to minimize their head motion. Prior to the MRI task, the participants engaged in a shock calibration procedure in which the participants themselves determined the intensity of the shocks (i.e., US). Further, they were instructed that they might learn to predict the occurrence of a shock if they attend to the depicted stimuli.
Behavioral performance measures	Average response times for risk ratings during the fMRI task were computed as a measure of information processing capacity and used as a covariate of no interest in several analyses.<br>Besides these performance measures, perceived risk of shock was assessed as the primary behavioral outcome.

Furthermore, we evaluated the German version of the STAI (STAI-T) as well as the German version of the Beck Depression Inventory II (BDI-II) and the Modified Fatigue Impact Scale (MFIS).

Acquisition

Imaging type(s)

Structural, Functional, Diffusion Weighted Imaging (DWI)

Field strength

3.0 Tesla (Siemens, Magnetom Prisma)

Sequence & imaging parameters

Structural: Acquisition of structural MRI scans comprised a saggital T1-weighted (T1w; 3-D-Magnetization Prepared Rapid Gradient Echo; MPRAGE) sequence with 208 slices encompassing the entire brain (0.8 mm3 isotropic voxels; TR = 2400 ms; TE = 2.22 ms; FA = 8°; FOV = 240 × 256 mm2; matrix size = 300 × 320; 5 min 8 s) and a sagittal FLAIR sequence (208 slices; 0.8 mm3 isotropic voxels; TR = 6000 ms; TE = 387 ms; TI = 2100 ms; FA = 120°; FOV = 240 × 256 mm2; matrix size = 300 × 320; 7 min 44 s duration).
Functional scans:T2*-weighted multi-band Echo-Planar-Imaging (EPI) Blood-Oxygen-Level-Dependent (BOLD) sequence from the Human Connectome Project; 2 × 2 × 2 mm3 isotropic voxels; TR = 800 ms; TE = 37 ms; flip angle = 52°; FOV = 208 mm × 208 mm; matrix size = 104 × 104; multi-band factor = 8
DWI scans: multi-shell DWI MRI sequence from the Human Connectome Project; 1.5 × 1.5 × 1.5 mm3 isotropic voxels; TR = 3230 ms; TE = 89.2 ms; flip angle = 78°; FOV = 210 · 210 mm2; matrix size 140 × 140; bandwidth = 1700 Hz/pixel; 2 shells [b = 1500 / 3000 s/mm2] ; 92 to 93 directions per shell; multi-band factor = 4, phase-encoding direction anterior-to-posterior)

Area of acquisition

Brain

Diffusion MRI ☒ Used ☐ Not used

Parameters

92 to 93 directions per shell; multi-band factor = 4, phase-encoding direction anterior-to-posterior, two shells [b = 1500 / 3000 s/mm2)

Preprocessing

Preprocessing software

Structural and functional MRI preprocessing and parts of the DWI preprocessing was conducted using SPM12 (https://www.fil.ion.ucl.ac.uk/spm/software/spm12/) and FSL (6.0.7.17; https://fsl.fmrib.ox.ac.uk/fsl/docs/#/). Remaining parts of DWI preprocessing were conducted with Mrtrix3 (https://www.mrtrix.org/).

Normalization

Structural and functional MRI normalization was conducted using SPM12 (i.e., spm_preproc_run.m). For DWI scans, we conducted an inverse normalization of the neuromorphometric brain atlas to the participants' individual DWI image space using the defomations toolbox included in SPM12.

Normalization template

Six-tissue type MNI-template distributed with SPM12 (i.e., "TPM.nii")

Noise and artifact removal

fMRI: Prior to normalization, the fMRI scans were corrected for inhomogeneity of the main magnetic field with FSL's topup algorithm and corrected for head motion using the SPM12 realignment procedure. After normalization, a spatial smoothing of fMRI scans was performed (8 mm full-width at half maximum Gaussian kernel) and a temporal high-pass filter (128 s cut-off) applied. Further, the voxel time series were corrected for head motion effects.
DWI: Prior to the anatomically constrained tractography conducted with Mrtrix3, the DWI scans were corrected for inhomogeneity of the main magnetic field using FSL's topup algorithm.

Volume censoring

None

Statistical modeling & inference

Model type and settings

fMRI: Two-step procedure. In the first step, a mass-univariate modeling of intra-participant fMRI voxel-timeseries with the General Linear Model (GLM) was performed. In the second, the resulting voxel-wise regression coefficients (either extracted from the brain's entire grey matter [GM] or GM in ROI included in a brain atlas) were used for prediction of perceived risk ratings.
In particular, in the first step, we modeled brain activity reflected by voxel timeseries during the two generalization runs per participant in a run-wise fashion with a design matrix including eight regressors reflecting the timing of task components, five of which were regressors of interest – one for each ring-shaped stimulus (i.e., the CS+, GS1 – 3, and the CSr-). In addition, it contained a regressor for the CSs-, one for trials where the CS+ was paired with the US, and one for the button presses performed to rate the risk of shock. Besides these regressors reflecting the timing of task components, another six regressors (derived from the realignment of fMRI scans during preprocessing) were included in the design matrix reflecting the participants' head motion. Except for the regressor coding button presses, boxcar regressors coding ones for the two-time bins per trial presenting CS or GS and zeros for the ITI time bins were determined first for the eight regressors reflecting task components. For button presses, the boxcar regressor coded ones for the period from the onset of the rating bin to the time of the button press and zero for the remaining time. After these boxcar regressors were defined, they were convolved with the hemodynamic response function to account for the temporal characteristics of the BOLD response. The full design matrix of all fourteen regressors was then GLM to model the neural responsivity to each regressor. Voxel-wise regression coefficient maps for each of the five regressors of interest per participant and generalization run were entered into the fMRI analyses as training and test patterns for a Support Vector Regresion (SVR) model. Based on the inclusion criteria defined for risk ratings and the availability of fMRI data, all 230 patterns were available for the 23 HPs, 130 for the 13 PwMSA and finally 305 for the 31 PwMSNA.
In the second step, we employed Support Vector Regression (SVR) to test our hypothesis that fear generalization recruits overlapping neural processing systems in HPs and PwMS with and without anxiety. Specifically, during training, an SVR

algorithm implemented in Matlab (MathWorks, Natick, Massachusetts, USA) learned to associate 230 fMRI fear response (i.e., regression coefficient) patterns of persons neither affected by MS nor anxiety (i.e., of HPs) with the associated perceived risk. Consistent with the approach in the analysis of behavioral fear generalization, perceived risk was computed by the subject-specific logistic regression models based on the ratings for the different ring-shaped stimuli. In testing, we used the HP-derived SVR model for predicting the true perceived risk (modeled by logistic regresion) based on the 130 voxel patterns derived from PwMSA and the 305 voxel patterns from PwMSNA. The correlation between true perceived risk and that predicted by SVR served as accuracy measure. Default SVR hyperparameters predefined by Matlab were used. Permutation testing (20,000 permutations of training labels) was used for statistical inference in one-sided tests. Bootstrapping tested the accuracies' robustness against (i.e., independence of) variations in the specific distribution of patterns and labels (1,000 resamplings of the HPs' training data) which was expressed in terms of the 95% accuracy confidence intervals (CI95%) computed across the 1,000 accuracies obtained per patient group. Potential effects of demographic and disease-related nuisance factors on accuracy were tested in the Supplement by repeating this analysis with fMRI patterns adjusted for covariates of no interest (CNI).
To evaluate the contribution of individual brain regions in both MS groups, we repeated the whole-brain GM analysis based on activity of coordinates located in the intersection of individual GM regions defined by the brain atlas and the GM group mask per atlas region. 10,000 permutations per region were performed for statistical inference (one-sided tests). The significance threshold was adjusted for family-wise error (FWE) with the Bonferroni method (i.e., by dividing the α-level for a single test [0.05] by the number of regions [i.e., 120] yielding 4.2 · 10-4). To evaluate regional neural substrates of altered fear processing in PwMSA, we tested for regions showing group differences in accuracy for PwMSA vs. PwMNSA using a Fisher Z-test and by appyling an FWE-corrected threshold.

DWI: ROI-wise. Specifically, a probabilistic Anatomically Constrained Tractography (ACT; 28) was performed using algorithms from Mrtrix3, FSL, and SPM12 on multi-shell multi tissue DWI scans. Specifically, we estimated diffusion basis functions from the individuals' multi-shell, multi-tissue DWI scans and computed fiber orientation densities, which were deconvolved with the basis functions using the constrained spherical deconvolution approach in Mrtrix3. We then created a GM/WM boundary map for each participant from their MPRAGE scans to define physiologically plausible regions as start and stop points for the streamline tracing algorithm in Mrtrix3. tThis boundary (segmented GM maps from the previous step) was coregistered to the participants' DWI scans, and ACT was performed. In this process, streamline length was limited to 250 mm, the fiber orientation distribution cutoff for streamline termination was set to 0.06, and 10 million streamlines were computed per participant.
We subsequently performed inverse normalization of the Neuromorphometrics brain atlas (defined in MNI space) to coregister it to the same space as the participants' DWI scans using SPM12. The structural connectivity matrix, reflecting the number of streamlines connecting pairs of regions in the coregistered Neuromorphometrics brain atlas, was computed using Mrtrix3.
These matrices were entered into the Brain Connectivity Toolbox to compute three regional connectivity measures of key importance to a wide range of brain disorders including MS for each participant and area in the Neuromorphometrics atlas. These measures included: the regional degree, the betweenness centrality, and the clustering coefficient. Multiple linear regression was finally used to compute (i) associations between these regional connectivity parameters and the patients' behavioral fear generalization parameters or (ii) differences in these parameters between PwMSA and PwMSNA.

Effect(s) tested

fMRI: Association between true and predicted perceived risk ratings assessed in terms of Pearson product moment correlation coefficients – either based on fMRI activity patterns extracted across all GM areas in the brain or based on GM areas in regions included in a brain atlas.
DWI: (i) Associations between graph-based structural connectivity measures (regional degree, betweenness centrality and clustering coefficient) on one hand and patients' behavioral fear generalization parameters computed for regions included in the brain atlas. (ii) Differences between these parameters between PwMSA and PwMSNA in a post-hoc analysis.

Specify type of analysis: ☐ Whole brain ☐ ROI-based ☒ Both

Anatomical location(s): Whole-brain grey matter; regions (ROI) included in the Neuromorphometrics atlas distributed with SPM12

Statistic type for inference (See Eklund et al. 2016): Entire grey matter-wise and ROI-wise

Correction: In the ROI-wise fMRI and DWI analyses, the Bonferroni correction was used to account for multiple testing (i.e., 120 tests were conducted, one per GM region included in the atlas).

Models & analysis

n/a	Involved in the study
☒	☐ Functional and/or effective connectivity
☐	☒ Graph analysis
☐	☒ Multivariate modeling or predictive analysis

Graph analysis: See Model type and setting for DWI.

Multivariate modeling and predictive analysis: See Model type and setting for fMRI.
